# F14. REDUCED DURATION MISMATCH NEGATIVITY ASSOCIATED WITH DECREASED GLUTAMATE+GLUTAMINE LEVEL IN SUBJECTS AT CLINICAL HIGH-RISK FOR PSYCHOSIS

**DOI:** 10.1093/schbul/sby017.545

**Published:** 2018-04-01

**Authors:** Yingying Tang, TianHong Zhang, Junjie Wang, LiHua Xu, Zhenying Qian, HuiRu Cui, Larry J Seidman, Robert W McCarley, Matcheri S Keshavan, William S Stone, Margaret Niznikiewicz, JiJun Wang

**Affiliations:** 1 Shanghai Mental Health Center, Shanghai Jiaotong University School of Medicine; 2 Harvard Medical School, Beth Israel Deaconess Medical Center; 3 Harvard Medical School, Veterans Affairs Boston Healthcare System, Beth Israel Deaconess Medical Center

## Abstract

**Background:**

Abnormal mismatch negativity (MMN), thought to be a putative marker of glutamatergic function, has been reported in non-Asian, first episode schizophrenia and clinical high-risk for psychosis (CHR) individuals as indicative of impairments in pre-attentive processes. However, reports of abnormal MMN in Asian populations are sparse, as well as its relationships to glutamate and γ–aminobutyric acid (GABA) levels in medial prefrontal cortex. The present longitudinal study explored MMN differences between CHR subjects who will and who will not remit, and its relationships with prefrontal glutamate and GABA levels.

**Methods:**

All subjects participated in the ShangHai At-Risk for Psychosis (SHARP) program. CHR subjects met the criteria defined by the Chinese version of the Structural Interview for Prodromal Syndromes (SIPS). From the SHARP sample, 76 CHR subjects (41 male, age 18.63 ± 5.02 years) and 53 HC (31 male, age 17.72 ± 3.18 years) completed both MMN test and proton magnetic resonance spectroscopy (1H MRS) scans using a MEGA-PRESS sequence at their initial visit. CHR subjects were divided into remitted (37) and non-remitted (34) individuals based on their clinical symptoms and functional scores at a one-year follow up. Duration MMN amplitude was measured at electrodes F1/2, Fz, FC1/2, FCz, C1/2 and Cz. Concentrations of glutamate+glutamine (Glx) and GABA in the medial prefrontal cortex (mPFC) were quantified using the LCModel software (version 6.3-0I).

Repeated measures analysis of variance (ANOVA) with group (remitted CHR, non-remitted CHR and HC) as the between-group factor and electrodes (Fz, FCz and Cz) as the within-group factor were performed for the midline sites, and the ANOVA using F1/2, FC1/2 and C1/C2 with laterality (left and right hemisphere) as an additional within-group factor was performed for the lateral sites. Correlations of the dMMN amplitude (averaged over the 9 electrodes) and Glx and GABA concentrations were assessed by Pearson correlation tests for each group.

**Results:**

There was a significant main effect of group (F(2,121)=3.14, p<0.05) for the midline fronto-central dMMN amplitude. Post-hoc tests showed that non-remitted CHR subjects had lower baseline dMMN amplitude (-4.75 ± 0.37μv) than HC (-5.92 ± 0.30μv, p<0.05), whereas dMMN in remitted CHR (-5.22 ± 0.36μv, p=0.41) was comparable to dMMN in HC.

The main effect of group was marginally significant at lateral sites (F(2,121)=2.83, p=0.06). DMMN amplitude in non-remitted CHR (-4.67 ± 0.37μv) tended to be lower than those in HC (-5.76 ± 0.29μv, p<0.1), while remitted CHR had dMMN amplitude (-5.11 ± 0.35μv, p=0.47) comparable to HC. There was no significant main effect of laterality or interaction of group × laterality.

In non-remitted CHR subjects, dMMN amplitude was significantly correlated with Glx level (r=-0.47, p<0.01) and with GABA level (r=-0.38, p<0.05) in the mPFC. However, the correlation of dMMN amplitude with Glx or GABA levels was not significant among either HC or remitted CHR.

**Discussion:**

In line with previous studies, reduced dMMN amplitude distinguished between remitted and non-remitted CHR subjects, with remitted CHR not different from HCs. Our finding further supports the idea that reduced dMMN amplitude could be a candidate biomarker for predicting outcome in CHR. More importantly, we linked the reduced dMMN amplitude in non-remitted CHR to their Glx and GABA levels in mPFC, the region identified as one of dMMN sources (responsible for attention switching) thus supporting the idea that NMDA-mediated disruptions may play a key role in predicting psychosis and functional outcome.

